# Retained organic solutes, patient characteristics and all-cause and cardiovascular mortality in hemodialysis: results from the retained organic solutes and clinical outcomes (ROSCO) investigators

**DOI:** 10.1186/1471-2369-14-134

**Published:** 2013-06-27

**Authors:** Michal L Melamed, Laura Plantinga, Tariq Shafi, Rulan Parekh, Timothy W Meyer, Thomas H Hostetter, Josef Coresh, Neil R Powe

**Affiliations:** 1 Departments of Medicine and Epidemiology & Population Health, Albert Einstein College of Medicine, 1300 Morris Park Avenue – Ullmann 615, Bronx, NY 10461, USA; 2 Department of Epidemiology, Emory University, Atlanta, GA, USA; 3 Department of Medicine, Johns Hopkins School of Medicine, Baltimore, MD, USA; 4 Departments of Medicine and Pediatrics, University of Toronto, Toronto, Canada; 5 Department of Medicine, VA Palo Alto HCS and Stanford University, Palo Alto, CA, USA; 6 Case Western University School of Medicine, Cleveland, OH, USA; 7 Departments of Epidemiology and Biostatistics, Johns Hopkins Bloomberg School of Public Health, Welch Center for Prevention, Epidemiology and Clinical Research, Johns Hopkins University, Baltimore, MD, USA; 8 Department of Medicine, University of California and Center for Vulnerable Populations, San Francisco General Hospital, San Francisco, CA, USA

**Keywords:** All-cause Mortality, Cardiovascular Mortality, Dialysis Outcomes, Indoxyl Sulfate, P-cresol Sulfate, Uremic Solutes

## Abstract

**Background:**

Multiple solutes are retained in uremia, but it is currently unclear which solutes are toxic. Small studies suggest that protein-bound solutes, such as p-cresol sulfate and indoxyl sulfate and intracellular solutes, such as methylamine (MMA) and dimethylamine (DMA), may be toxic. Our objective was to test whether elevated levels of these solutes were associated with mortality.

**Methods:**

We conducted a prospective cohort study in 521 U.S. incident hemodialysis patients to evaluate associations between these solutes and all-cause and cardiovascular mortality. P-cresol sulfate, indoxyl sulfate, MMA and DMA levels were measured from frozen plasma samples obtained 2 to 6 months after initiation of dialysis. Mortality data was available through 2004 using the National Death Index.

**Results:**

Elevated (greater than the population median) p-cresol sulfate, MMA or DMA levels were not associated with all-cause or cardiovascular mortality. Elevated indoxyl sulfate levels were associated with all-cause mortality but not cardiovascular mortality (hazard ratio 1.30 (95% confidence interval 1.01, 1.69) p-value 0.043).

**Conclusions:**

In this cohort of 521 incident hemodialysis patients, only elevated indoxyl sulfate levels were associated with all-cause mortality. Further research is needed to identify causes of the toxicity of uremia to provide better care for patients with kidney disease.

## Background

There are currently nearly 400,000 patients with end-stage renal disease (ESRD) living on dialysis in the US [1]. These patients experience a high degree of morbidity and mortality, with a first-year mortality rate of almost 20% [1]. More than 50% of the deaths of hemodialysis patients in the US are attributed to cardiovascular disease [2], but both cardiovascular and non-cardiovascular death risks are elevated relative to the general population [3]. Current guidelines for dialysis adequacy are based on the removal of urea, a substance that is elevated in ESRD but has not been shown to be toxic when it accumulates in animals. In addition to urea, there are over 200 other solutes with different characteristics that are retained in ESRD patients treated with hemodialysis [4,5]. Some of these potential putative toxins include the protein-bound solutes p-cresol sulfate and indoxyl sulfate and intracellularly sequestered methylamine (MMA) and dimethylamine (DMA), which are not removed efficiently by conventional hemodialysis techniques [6,7].

Studies have shown p-cresol sulfate and indoxyl sulfate to be associated with all-cause mortality, cardiovascular events and endothelial dysfunction in patients with kidney disease [8-12]. Epidemiological studies of MMA and DMA have been fewer, but mouse studies demonstrated that these compounds may be anorectic agents [13-15]. Due to the smaller nature of previous investigations and varying dialysis practices in Europe, we tested the associations between these retained organic solutes and cardiovascular and all-cause mortality in a relatively large, well-characterized cohort of US hemodialysis patients.

## Methods

### Study population

Our study population was a sub-cohort of the CHOICE Study. CHOICE was a national, prospective cohort study investigating dialysis treatment choices and patient outcomes in incident end-stage renal disease (ESRD) patients. A total of 1041 dialysis patients (767 hemodialysis and 274 peritoneal dialysis) were enrolled in the USA from 81 dialysis clinics in 19 states between October 1995 and June 1998. These included clinics associated with Dialysis Clinic, Inc. (DCI, Nashville, TN; n = 923), New Haven CAPD (New Haven, CT; n = 86) and St. Raphael’s Hospital (New Haven, CT; n = 32). Entry criteria included the initiation of chronic outpatient dialysis in the preceding 3 months, ability to provide informed consent for participation, age older than 17 years, and ability to speak English or Spanish.

A specimen bank was established to store blood samples only from the DCI participants, and specimens were obtained for 898 (97.3%) of the DCI enrollees. The Johns Hopkins University School of Medicine Institutional Review Board (IRB) and the review boards for the clinical centers approved the study protocol. The Albert Einstein College of Medicine, Stanford University, Johns Hopkins University School of Medicine and the University of California at San Francisco IRBs all approved the protocol detailed in this analysis. All patients gave written informed consent before participation in the study. For the current study, we restricted analysis to hemodialysis patients.

### Data collection

Plasma from special draws was available for 585 patients (81% of all specimen bank participants treated with hemodialysis) from 2 to 33 months after dialysis initiation, with 521 patients having a sufficient volume (at least 1 ml) of plasma within 6 months of dialysis initiation. We measured uremic solutes in stored plasma from these 521 CHOICE participants with available special draw samples within 6 months of dialysis initiation. Detailed methods for the CHOICE special blood draw have been previously described [16]. Briefly, a lavender-top tube (EDTA) was collected pre-dialysis between 1996 and 1998, immediately centrifuged at 2500–3000 rpm for 15 minutes, separated and refrigerated in the dialysis unit then shipped on ice and stored at −80° Celsius at the DCI central laboratory (Nashville, TN). Specimens for this study were thawed and divided into two equal aliquots at the central laboratory, mailed by overnight courier on dry ice to the two study laboratories (Stanford University and the Albert Einstein College of Medicine), and then stored at −80° Celsius at the respective laboratories until they were thawed for analysis. Measurement of p-cresol sulfate and indoxyl sulfate was performed at Stanford University and methylamine and dimethylamine at the Albert Einstein College of Medicine.

### Measurement of solutes

**Figure 1:**
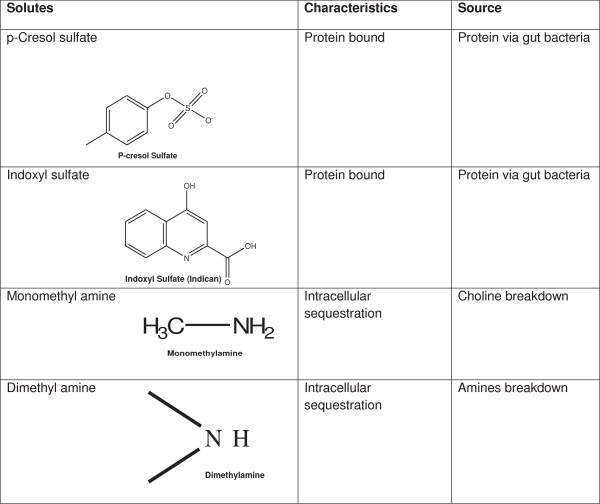
Organic solutes and their characteristics and sources.

P-cresol sulfate (Figure 1) was assayed by HPLC as previously described with fluorescence detection at excitation 214 nm and emission 306 nm [17]. Recovery of p-cresol sulfate with the assay was 100 ± 1% of p-cresol sulfate added to normal plasma to achieve concentrations similar to those found in patients on dialysis (n = 4), and repeat p-cresol sulfate assay of plasma from dialysis patients after freeze-thaw yielded 94 ± 7% of the original value (n = 6). Coefficient of variation was 0.51%.

Indoxyl sulfate (Figure 1) was measured as previously described [17] on the same column as p-cresol sulfate with fluorescence detection using excitation 295 nm and emission 390 nm. Recovery with the assay was 100 ± 1 percent of indoxyl sulfate added to normal plasma to achieve concentrations similar to those found in patients on dialysis (n = 4) and repeat assay of after freeze-thaw yielded 97 ± 10 percent of the original value (n = 6). Coefficient of variation was 0.60%.

MMA and DMA (Figure 1) levels were measured by chromatography using a Shimadzu Prominence HPLC system that consisted of two LC-20 AD pumps, a DGU-20A3 degasser, a SIL-20A auto sampler, and an RF-10AXL fluorescence detector. Determinations were duplicated. Peak area was used for quantification [18]. Coefficients of variation were 0.44% for MMA and 0.24% for DMA.

### Outcomes

#### All-cause mortality

Mortality was ascertained primarily through linked National Death Index (NDI) data, in addition to communication with the dialysis clinics, monthly reports from the central DCI database, and linked Centers for Medicare and Medicaid Services (CMS) data.

#### Cardiovascular mortality

Atherosclerotic cardiovascular disease (ASCVD) was assigned as the cause of death in CHOICE through two sources of information: adjudicated death records and National Death Index (NDI) records. For adjudicated death records, medical charts were reviewed by a Cardiovascular Disease Endpoints committee of trained physicians using uniformly applied criteria modified from the Cardiovascular Health Study [19] and the HEMO study [20]. Each chart was independently reviewed by two members of the committee and final immediate and underlying causes of death were verified by an independent adjudicator. Adjudicated causes of death were determined to be ASCVD if either the immediate or underlying cause of death was related to ischemic heart disease, cerebrovascular disease, or peripheral vascular disease, including abdominal aortic aneurysm and ischemic bowel disease.

NDI records were obtained through 2004 for CHOICE patients. These records provided cause of death information (ICD-9/ICD-10 codes) from the U.S. Standard Certificate of Death. All codes in Section 27, Part I (including immediate and underlying causes of death) were included, whereas codes in Part II (contributing causes) were not. Any code for coronary artery disease [ICD-9: 410, 411, 414.0, 414.8, 414.9, 429.2; ICD-10: I21, I22, I24, I25.0, I25.1, I25.5, I25.6, I25.8, I25.9, I51.6], cerebrovascular disease [ICD-9: 431, 432.9, 433, 434, 436, 437.0, 437.1, 437.8, 437.9; ICD-10: I61, I62.9, I63, I64, I67.2, I67.9], abdominal aortic aneurysm [ICD-9: 441; ICD-10, I71], peripheral vascular disease [ICD-9: 443.9, 785.4; ICD-10: I73.9, R02], or ischemic bowel [ICD-9: 557; ICD-10: K55] was considered to be ASCVD-related.

ASCVD death was assigned in CHOICE if either the adjudicated death record or NDI records indicated that cause of death was ASCVD-related.

### Covariates

Data regarding patient demographics and medical history were collected from a self-report questionnaire and chart review. Race was self-categorized and coded as African-American or white/other (including Hispanic, Asian and Native American). The degree of comorbidity severity was assessed using the Index of Coexistent Disease (ICED), an instrument that has been validated in dialysis populations [21]. The composite ICED score ranged from 0 to 3 (with 3 indicating highest severity) and was calculated from two constituent indices, the 19-axis Index of Disease Severity (IDS) and the 11-axis Index of Physical Impairment (IPI). Gastrointestinal disease included a history of or active esophagitis, gastritis, ulcers, pancreatitis, colitis, hiatal hernia, diabetic gastroparesis, reflux, diverticulosis, polyps, hemorrhage or perforation. Residual renal function was obtained from the baseline self-report questionnaire and was defined as the ability to produce at least 250 mL of urine daily. Albumin and creatinine were measured as part of routine labs and values in the 3-month window surrounding baseline were averaged. High-sensitivity C-reactive protein (hs-CRP) levels were measured in all patients with frozen serum available in the CHOICE specimen bank a median 5 months from dialysis initiation [16].

### Statistical analysis

We first compared patient characteristics by levels of retained solutes (divided at the median for each solute) using Pearson’s χ^2^ tests for categorical variables and ANOVA for continuous variables. In order to try to get a better estimate of total solute burden, we created a score for the number of solutes above the median for each patient. The score ranged from 0, if all solutes were below the median value, to 4, if all solute levels were above the median value. Pearson’s correlations were performed to evaluate correlations between solute levels and serum creatinine levels.

We used Cox proportional hazards analysis to assess the presence, strength, independence and statistical significance of the association between individual solute levels above vs. below the median value and cardiovascular and all-cause mortality. Modeling building included potential confounders that were associated with the predictors and/or outcomes in univariate models. Potential confounders included in the model were age at enrollment, sex, race/ethnicity, comorbidity score (ICED), and baseline albumin. Another model adjusted for all of the above with the addition of obesity and baseline serum creatinine values. Models were also stratified by potential effect modifiers such as diabetes, albumin, and gastrointestinal disease. Gastrointestinal disease was considered because p-cresol sulfate and indoxyl sulfate are produced by gut bacteria. Patients were censored at transplantation or last date of follow-up (December 31, 2004). Sensitivity analyses were performed with all-cause mortality updated to 12/31/2008. All analyses were stratified by clinic to account for possible inter-clinic variability. The threshold for statistical significance was set at a two-sided p-value of < 0.05. Statistical analyses were performed using Stata software, version 11.2 (Stata Corporation, College Station, TX).

## Results

### Characteristics of the study population and retained solute levels

**Figure 2:**
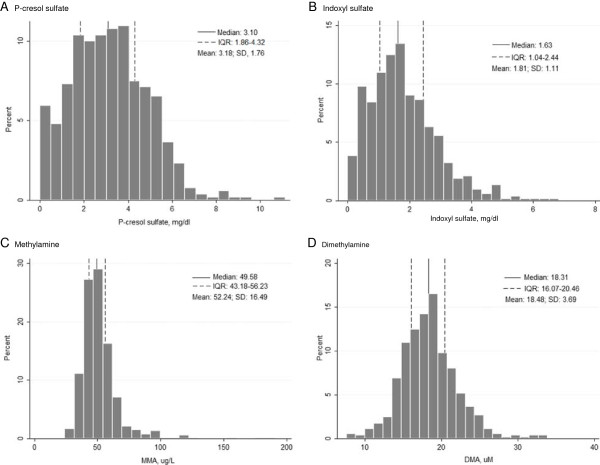
**Distribution of measured solutes.** Solid lines, median values; dashed lines, interquartile range (IQR). **A**. P-cresol sulfate, **B**. Indoxyl sulfate, **C**. Methylamine, **D**. Dimethylamine.

Overall there were 521 participants with available plasma for analysis. Their mean age was 58.3 years, 61% were white, 54% were male and 39% had residual urine output (defined as >250 ml/day) after one year of dialysis (Table 1). Choices for Healthy Outcomes in Caring for End-Stage Renal Disease (CHOICE) hemodialysis patients who were excluded due to measurement >6 months after start of dialysis or insufficient or missing plasma did not differ from the study population, except for being slightly older (61.5 vs. 58.3 years, *P* = 0.005) and sicker (36.9% vs. 29.3% with severe comorbidity, *P* = 0.04). The distributions of the solutes are shown in Figure 2. Higher p-cresol sulfate (Table 1) levels were associated with older age, male sex, lower BMI, higher mean albumin and lower median CRP levels. Higher indoxyl sulfate levels were associated with lower ICED scores and higher mean serum albumin and creatinine levels. Higher MMA levels were associated with older age, higher mean serum albumin and creatinine. Higher DMA levels were associated with a higher mean serum creatinine. Levels of solutes were generally positively but weakly correlated with levels of serum creatinine and each other (Table 2).

**Table 1:** Characteristics of study participants by levels of solutes

**Characteristic**	**Overall**	**P-cresol sulfate (mg/dl)**		**Indoxyl sulfate (mg/dl)**		**Monomethylamine (μg/l)**		**Dimethlyamine (μM)**	
		**Low**	**High**	**Low**	**High**	**Low**	**High**	**Low**	**High**
		***0.0-3.l***	***3.1-11.1***	***0-1.6***	***1.6-6.8***	***24.2-49.6***	***49.7-190.6***	***7.6-18.3***	***18.3-33.9***
*N (%)*	*521 (100%)*	261 (50.1)	260 (49.9)	261 (50.1)	260 (49.9)	262 (50.3)	259 (49.7)	263 (50.5)	258 (49.5)
**Demographics**									
Mean age (SD)	58.3 (14.7)	56.6 (14.2)*	59.9 (14.9)*	57.3 (13.9)	59.2 (15.3)	56.9 (15.2)*	59.6 (14.0)*	58.6 (14.9)	57.9 (14.4)
% white	61.0	61.7	60.4	59.0	63.1	63.4	58.7	63.5	58.5
% male	54.1	47.1*	61.2*	54.4	53.9	50.0	58.3	53.6	54.7
**Clinical**									
% ICED = 3	29.2	31.4	26.9	35.6*	22.7*	27.1	31.3	26.2	32.2
Mean BMI (SD)	27.4 (7.0)	28.3 (7.4)*	26.5 (6.5)*	27.7 (7.3)	27.1 (6.6)	27.5 (7.7)	27.3 (6.2)	27.1 (7.4)	27.8 (6.5)
% with residual urine output at 1 year	38.9	41.4	36.6	42.9	35.2	37.4	40.4	42.3	35.6
Mean KtV (SD)	1.27	1.26	1.29	1.24*	1.31*	1.27	1.27	1.27	1.27
Cause of ESRD									
Diabetes mellitus	47.8	50.2*	45.4*	56.7**	38.9**	49.2	46.3	46.4	49.2
Hypertension	18.2	13.0*	23.5*	14.9**	21.5**	16.0	20.5	19.4	17.1
Glomerulonephritis	16.1	18.4*	13.9*	10.3**	21.9**	14.5	17.8	15.2	17.1
Other	17.9	18.4*	17.3*	18.0**	17.7**	20.2	15.4	15.2	17.1
% with diabetes	54.9	56.7	53.1	63.2**	46.5**	55.7	54.1	53.6	56.2
% with hypertension	97.0	96.5	97.6	96.6	97.5	97.5	96.6	96.5	97.6
% with gastrointestinal disease	41.8	44.8	38.9	43.3	40.4	39.7	44.0	42.6	41.1
**Laboratory**									
Mean albumin (SD)	3.6 (0.4)	3.6 (0.4)*	3.7 (0.3)*	3.6 (0.4)**	3.7 (0.3)**	3.6 (0.4)*	3.7 (0.3)*	3.7 (0.3)	3.6 (0.4)
Mean creatinine (SD)	7.3 (2.4)	7.1 (2.5)	7.5 (2.3)	6.5 (2.1)**	8.1 (2.5)**	6.9 (2.4)**	7.8 (2.4)**	6.9 (2.4)**	7.7 (2.4)**
Median CRP (IQR)	0.4 (0.2-1.1)	0.4 (0.2-1.5)*	0.4 (0.2-0.8)*	0.5 (0.2-1.2)	0.4 (0.2-1.0)	0.4 (0.2-1.2)	0.4 (0.2-1.0)	0.4 (0.2-1.3)	0.4 (0.2-1.0)

Low, below population median value for solute; high, at or above population median values for solute. Diabetes, hypertension, and gastrointestinal disease were defined by domains of the index of disease severity scale of the ICED. *n* = 521 for all variables except BMI (*n* = 492), albumin (*n* = 515), creatinine (*n* = 516), CRP (*n* = 517), Kt/V (*n* = 401) and residual urine output at 1 year (*n* = 447).

*P < 0.05 for high vs. low level of solute, by ANOVA or χ^2^ test.

**P < 0.001 for high vs. low level of solute, by ANOVA or χ^2^ test.

**Table 2:** Correlations between serum creatinine and retained solutes: p-cresol sulfate, indoxyl sulfate, methylamine and dimethylamine

**Solute**	**Serum creatinine**	**p-cresol sulfate**	**Indoxyl sulfate**	**Methylamine**	**Dimethylamine**
Serum creatinine	1.00				
p-cresol sulfate	0.10	1.00			
	0.02				
Indoxyl sulfate	0.41	0.33	1.00		
	< 0.001	< 0.001			
Methylamine	0.17	0.12	0.18	1.00	
	< 0.001	0.009	< 0.001		
Dimethylamine	0.16	0.10	0.19	0.13	1.00
	< 0.001	0.02	< 0.001	0.002	

Data presented by cell: upper part of cell: ρ, lower part of cell: p-value.

### Associations between retained solute levels and all-cause mortality

Three hundred thirty-three individuals died over the course of the study. There were no statistically significant associations between elevated solute levels and all-cause mortality except for the hazard ratio for indoxyl sulfate in the fully-adjusted model (hazard ratio (HR) 1.30 (95% confidence intervals (CI): 1.01, 1.69) (Table 3). The number of solutes with values above the median was not associated with all-cause mortality. The lack of association did not change when stratifying the population by baseline albumin, creatinine or the presence of gastrointestinal disease (data not shown). Additionally, the extension of all-cause mortality data through 2008 did not change the associations between solute levels and mortality (data not shown).

**Table 3:** **Relative hazards (95% CI) for*all-cause*mortality by solute**

**Solute**	**Overall (continuous, per 1 SD)**	**Hazard ratio (95% CI)**			
		**Below median**		**Above median**	
**P-cresol sulfate**					
*Range of values*	*0-11.1 mg/dl (SD = 1.76 mg/dl)*	*0-3.1 mg/dl*		*3.1-11.1 mg/dl*	
Unadjusted	1.05 (0.93-1.17)	Reference		1.10 (0.88-1.37)	
Adjusted*	1.02 (0.90-1.16)	Reference		1.03 (0.81-1.31)	
+ obesity, creatinine	1.02 (0.90-1.16)	Reference		1.03 (0.83-1.28)	
**Indoxyl sulfate**					
*Range of values*	*0-6.8 mg/dl (SD = 1.11 mg/dl)*	*0-1.6 mg/dl*		*1.6-6.8 mg/dl*	
Unadjusted	0.93 (0.84-1.04)	Reference		1.03 (0.83-1.28)	
Adjusted*	0.98 (0.87-1.10)	Reference		1.14 (0.90-1.44)	
+ obesity, creatinine	1.04 (0.91-1.18)	Reference		1.30 (1.01-1.69)	
**MMA**					
*Range of values*	*24.2-190.6 ug/L (SD = 16.48 ug/L)*	*24.2-49.6 ug/L*		*49.7-190.6 ug/L*	
Unadjusted	1.10 (0.99-1.23)	Reference		1.01 (0.81-1.26)	
Adjusted*	1.05 (0.94-1.17)	Reference		0.94 (0.74-1.20)	
+ obesity, creatinine	1.09 (0.97-1.22)	Reference		1.00 (0.78-1.29)	
**DMA**					
*Range of values*	*7.6-33.9 uM (SD = 3.69 uM)*	*7.6-18.3 uM*		*18.3-33.9 uM*	
Unadjusted	0.96 (0.86-1.07)	Reference		0.86 (0.69-1.08)	
Adjusted*	1.00 (0.89-1.12)	Reference		0.88 (0.69-1.11)	
+ obesity, creatinine	1.01 (0.90-1.14)	Reference		0.91 (0.71-1.17)	
Total Score (vs. 0)**	**Continuous**	**1**	**2**	**3**	**4**
Unadjusted	1.00 (0.91-1.09)	1.53 (1.03-2.27)	1.24 (0.87-1.77)	1.21 (0.83-1.77)	1.14 (0.72-1.79)
Adjusted*	0.99 (0.89-1.10)	1.28 (0.85-1.94)	1.24 (0.85-1.80)	0.96 (0.64-1.43)	1.26 (0.78-2.04)
+ obesity, creatinine	1.04 (0.93-1.16)	1.29 (0.84-1.99)	1.28 (0.86-1.90)	1.06 (0.69-1.64)	1.49 (0.88-2.51)

All models stratified by clinic cluster. *Adjustment: basic model, age at enrollment, sex, race, comorbidity score (ICED), and baseline albumin: + obesity and creatinine value. **Total number of solutes with levels above the population median.

### Associations between retained solute levels and cardiovascular mortality

There were no associations between elevated solute levels and cardiovascular mortality in either unadjusted or multivariable-adjusted models (Table 4). The unadjusted estimate for p-cresol sulfate was elevated HR 1.30 (95% CI: 0.89, 1.89) but did not reach statistical significance. There was also no association in multivariable adjusted models between cardiovascular mortality and the number of solutes above the median. The lack of association did not change when stratifying the population by baseline albumin, creatinine or the presence of gastrointestinal disease (data not shown).

**Table 4:** **Relative hazards (95% CI) for*cardiovascular*mortality by solute**

**Solute**	**Overall (continuous, per 1 SD)**	**Hazard ratio (95% CI)**			
		**Below median**		**Above median**	
**P-cresol sulfate**					
*Range of values*	*0-11.1 mg/dl (SD = 1.76 mg/dl)*	*0-3.1 mg/dl*		*3.1-11.1 mg/dl*	
Unadjusted	1.09 (0.93-1.28)	Reference		1.35 (0.98-1.85)	
Adjusted*	1.06 (0.89-1.27)	Reference		1.24 (0.87-1.77)	
+ obesity, creatinine	1.07 (0.88-1.29)	Reference		1.30 (0.89-1.89)	
**Indoxyl sulfate**					
*Range of values*	*0-6.8 mg/dl (SD = 1.11 mg/dl)*	*0-1.6 mg/dl*		*1.6-6.8 mg/dl*	
Unadjusted	0.87 (0.74-1.03)	Reference		0.93 (0.67-1.27)	
Adjusted*	0.88 (0.74-1.06)	Reference		0.93 (0.66-1.31)	
+ obesity, creatinine	0.96 (0.79-1.18)	Reference		1.11 (0.76-1.64)	
**MMA**					
*Range of values*	*24.2-190.6 ug/L (SD = 16.48 ug/L)*	*24.2-49.6 ug/L*		*49.7-190.6 ug/L*	
Unadjusted	1.06 (0.90-1.24)	Reference		0.99 (0.72-1.36)	
Adjusted*	0.97 (0.82-1.15)	Reference		0.91 (0.64-1.29)	
+ obesity, creatinine	1.06 (0.89-1.26)	Reference		1.01 (0.69-1.48)	
**DMA**					
*Range of values*	*7.6-33.9 uM (SD = 3.69 uM)*	*7.6-18.3 uM*		*18.3-33.9 uM*	
Unadjusted	0.97 (0.83-1.14)	Reference		0.81 (0.58-1.13)	
Adjusted*	1.00 (0.85-1.18)	Reference		0.84 (0.59-1.18)	
+ obesity, creatinine	1.02 (0.86-1.22)	Reference		0.89 (0.61-1.29)	
Total Score (vs. 0)**	**Continuous**	**1**	**2**	**3**	**4**
Unadjusted	1.00 (0.88-1.14)	1.22 (0.70-2.13)	1.00 (0.61-1.65)	0.78 (0.45-1.35)	1.43 (0.79-2.59)
Adjusted*	0.97 (0.84-1.13)	1.03 (0.57-1.84)	0.89 (0.52-1.51)	0.59 (0.33-1.07)	1.37 (0.73-2.58)
+ obesity, creatinine	1.05 (0.89-1.25)	1.11 (0.60-2.06)	0.97 (0.55-1.71)	0.72 (0.38-1.37)	1.91 (0.94-3.88)

All models stratified by clinic cluster. *Adjustment: basic model, age at enrollment, sex, race, comorbidity score (ICED), and baseline albumin: + obesity and creatinine value. **Total number of solutes with levels above the population median.

## Discussion

In this national cohort of incident hemodialysis patients we found no associations between elevated plasma levels of total p-cresol sulfate, MMA or DMA and all-cause or cardiovascular mortality. We did find an elevated risk of all-cause mortality with higher indoxyl sulfate levels but only in the fully-adjusted model. While this finding is similar to previous research detailed below, in the setting of multiple comparisons in the current analysis and the borderline p-value (0.043), we are cautious with the significance of this finding. The search for uremic toxins has been ongoing in the nephrology community for many years [22]. The use of urea measurement alone is a crude measure of uremia that does not reflect the multitude of solutes that accumulate during renal failure and contribute to disease. Discovery of toxic solutes that accumulate in renal failure and could be diminished by alterations in dialysis or by reduction in their production would allow for tailoring of dialysis therapy and ultimately improve patient outcomes. For example, protein-bound solutes such as p-cresol sulfate and indoxyl sulfate can be removed more effectively by increasing dialyzer surface area and dialysate flow [23]. Similarly, intracellularly sequestered compounds such as MMA would be expected to be removed best by longer dialysis sessions.

The solute p-cresol sulfate has had the most observational data to suggest toxicity. While our study showed a borderline significant association between elevated p-cresol sulfate and cardiovascular mortality, it disappeared with adjustment for other factors including residual renal function and baseline co-morbidities. In a cohort of 139 patients with different stages of CKD (ranging from stage 2 to stage 5 on dialysis), baseline free and total p-cresol sulfate showed an inverse relationship with renal function and a positive relationship with vascular calcification [10]. This study, which included only Caucasian prevalent CKD patients, measured free and total p-cresol sulfate [10]. They also found that free but not total p-cresol sulfate levels were associated with mortality [10]. This group, using the same Caucasian cohort, showed that elevated levels of indoxyl sulfate were also associated with overall and cardiovascular mortality [9]. Another study in 100 prevalent hemodialysis patients in Taiwan also showed an association between free and total p-cresol with cardiovascular events [8]. A study of 175 prevalent hemodialysis patients in Belgium showed an elevated risk of all-cause mortality for patients with high free p-cresol sulfate levels (HR 2.28; 95% CI: 1.12, 4.64) [24]. A later study from the same group in Belgium showed that in 499 patients with mild to moderate kidney disease, higher baseline free p-cresol levels were associated with cardiovascular events (HR 1.39, p = 0.04) [11]. Most of the previous studies were limited by small sample sizes with few events and, thus, an inability to fully adjust in statistical models. Ours is also the only US based study evaluating these associations with differing patient populations, medications, dialysis practice patterns and lastly outcomes (CV mortality versus CV events). These differences compared to previous studies may partially explain the differential results.

Our study measured total levels of the solutes. Assays of total levels of protein-bound solutes may not reflect free levels due to variations in albumin concentration and the extent of protein binding. Other studies have shown that free but not total levels show stronger associations with mortality [10]. However, stratification of our results by albumin status did not show differential effects. Our study also used specimens from 2 to 6 months after dialysis initiation. The ideal timing of specimen collection for this type of study is unknown. Collecting samples before the initiation of dialysis may lead to more confounding by residual renal function, whereas samples from later in the course of dialysis may lead to survival bias, as not all the participants who started on dialysis will have survived to specimen collection. The uremic toxins we studied are all poorly cleared by current dialysis therapies and by the dialysis therapies used at the initiation of the CHOICE study, therefore, one would not expect large changes in levels after dialysis initiation, but this has never been studied.

Among patients receiving conventional dialysis, we demonstrated that protein-bound uremic solutes were not independently associated with mortality in a well-dialyzed cohort. This finding was consistent among all the uremic solutes and did not differ by diabetic or nutritional status. Our finding are similar to other studies evaluating associations between potential uremic toxins and mortality which also had null results [25]. Recent trials comparing more frequent or nocturnal daily dialysis have shown considerable improvement in outcomes, which has been attributed to improved dialysis and clearance [26,27]. The quality indicators such as lower blood pressure, hospitalization rates, and mortality, however, may all be related to improved volume, electrolytes and blood pressure, rather than greater removal of uremic solutes. This hypothesis still needs to be tested in the recently completed trials with both cardiovascular and mortality end-points.

We have focused only on major clinical events, mortality and CVD mortality. However, other disabilities suffered by patients receiving nominally adequate dialysis, such as neurocognitive and sleep disorders, quality of life indicators, response to medication such as ESAs and malnutrition that may benefit from increased solute clearance but further work is needed to test the relation of these non-urea solutes to such outcomes. Identification of solutes or classes of solutes associated with mortality and morbidiy could also lead to strategies for reducing their production or enhancing their elimination.

There are several limitations to our study. The first is that we did not have data on the entire CHOICE hemodialysis cohort, which may have introduced an unmeasured selection bias, since only those who survived and had the correct sample in the specimen bank were included. Our final cohort had less severe comorbid illness and early mortality than the overall CHOICE sample, in part because some specimens were obtained later in patients’ course of dialysis therapy. Another limitation is the use of plasma from a single time point as the exposure. Unfortunately, most studies of uremic solutes have had this limitation. Finally, like all previous studies on these potential uremic toxins, our study is observational and therefore causality cannot be directly inferred. These limitations are balanced by the several strengths of our study, including prospective design with inclusion of only incident hemodialysis patients; detailed and precise information for demographic, clinical and treatment factors; and systematic adjudication of baseline comorbid conditions as well as outcomes. These comprehensive data allowed us to extensively adjust for potential confounders in our analysis.

## Conclusions

In summary, in this large national incident dialysis cohort, baseline levels of total p-cresol sulfate, MMA, and DMA were not associated with all-cause or cardiovascular mortality. Elevated levels of indoxyl sulfate did show an association with all-cause mortality but not cardiovascular mortality and not in all models. Studies in the future should focus on longitudinal measurements and on other putative uremic toxins, including asymmetric dimethyl arginine (ADMA) and others. Further research is warranted in this area in order to try to improve dialysis patient outcomes.

## Competing interests

The authors declare that they have no competing interests.

## Authors’ contributions

THH and TWM performed the assays. MLM, LP, THH, TWM, JC, RP, TS and NRP conceived of the study and participated in its design and coordination. LP performed the statistical analysis. MLM drafted the manuscript. All authors read, made meaningful changes to and approved the final manuscript.

## Pre-publication history

The pre-publication history for this paper can be accessed here:

http://www.biomedcentral.com/1471-2369/14/134/prepub
